# High Urban Breeding Densities Do Not Disrupt Genetic Monogamy in a Bird Species

**DOI:** 10.1371/journal.pone.0091314

**Published:** 2014-03-10

**Authors:** Sol Rodriguez-Martínez, Martina Carrete, Séverine Roques, Natalia Rebolo-Ifrán, José L. Tella

**Affiliations:** 1 Department of Biology, Biochemistry and Pharmacy, Universidad Nacional del Sur, Bahía Blanca, Argentina; 2 Department of Conservation Biology, Estación Biológica de Doñana, CSIC, Sevilla, Spain; 3 Department of Physical, Chemical and Natural Systems, Universidad Pablo de Olavide, Sevilla, Spain; 4 Department of Ecology, Genetics and Evolution, IEGEBA-CONICET, Facultad de Ciencias Exactas y Naturales, Universidad de Buenos Aires, Buenos Aires, Argentina; University of Massachusetts, United States of America

## Abstract

Urbanization causes widespread endangerment of biodiversity worldwide. However, some species successfully colonize cities reaching higher densities than in their rural habitats. In these cases, although urban city dwellers may apparently be taking advantage of these new environments, they also face new ecological conditions that may induce behavioural changes. For example, the frequency of alternative reproductive behaviours such as extra-pair paternity and intraspecific brood parasitism might increase with breeding densities. Here, using a panel of 17 microsatellites, we tested whether increments in breeding densities such as those associated with urban invasion processes alter genetic monogamy in the burrowing owl *Athene cunicularia*. Our results show low rates of extra-pair paternity (1.47%), but relatively high levels of intraspecific brood parasitism (8.82%). However, we were not able to detect differences in the frequency at which either alternative reproductive behaviour occurs along a strong breeding density gradient. Further research is needed to properly ascertain the role of other social and ecological factors in the frequency at which this species presents alternative reproductive strategies. Meanwhile, our results suggest that genetic monogamy is maintained despite the increment in conspecific density associated with a recent urban invasion process.

## Introduction

Density affects direct interactions, both cooperative and competitive, by increasing spatial proximity among individuals. The spatial distribution of mates may, for example, influence the encounter rate between individuals, thus altering the frequency at which alternative reproductive behaviours such as extra-pair paternity or intraspecific brood parasitism appear [1]. Several studies have emphasized that many monogamous passerine birds, in which extra-pair paternity is relatively common [2], can show higher extra-pair copulation rates, and thus extra-pair paternity, as a consequence of increments in density [3]. However, extra-pair paternity is less common in non-passerine birds and its variability within and among species is still not fully understood [4]. Regarding intraspecific brood parasitism, there are examples of density-dependent changes in the frequency of this behaviour in a few bird species [5], [6], although surprisingly it is a poorly explored reproductive behaviour overall [7].

Urbanization is considered as one of the most severe and lasting forms of land-use modification that occurs unchecked worldwide [8], intensifying the current biodiversity crisis [9], [10]. However, the relationship between urbanization and biodiversity is multifaceted and complex, as species vary in their ability to respond to the drastic changes taking place along the urban-rural gradient [8], [11]. Indeed, although most species decline and go extinct in urbanized landscapes [12], others are able to colonize and even increase their densities in these human-modified areas [11], [13]. In these cases, although urban city dwellers may apparently be taking advantage of these new environments, they may also face new ecological conditions that can induce behavioural changes [14]–[16]. For example, novel selection pressures associated with urban environments may alter the rates of alternative reproductive strategies in birds [17], [18].

Here we investigate the frequency of alternative reproductive behaviours (extra-pair paternity and intraspecific brood parasitism) in the burrowing owl *Athene cunicularia* along a breeding density gradient associated with a recent invasion of urban habitats [11]. The burrowing owl is a socially monogamous territorial species widely distributed throughout North and South America, where it shows marked differences in population trends. In the northern hemisphere, the transformation of grasslands and the use of contaminants seems to be leading to a negative population trend [19]. By contrast, in South America, it is a relatively common species in areas with different levels of grazing pressure [20] and, in recent years, in urban environments [11]. The abundance varies there between neighbouring urban and rural habitats in an Argentinean population, with higher densities in the former compared to the latter [11]. Thus, under the density hypothesis, we would expect a higher frequency of extra-pair paternity and/or intraspecific brood parasitism in territories located closer to others and in highly populated areas. Our genetic results, obtained after analysing a large microsatellite panel (17 out of 23 available microsatellites), show that increments in breeding density are not promoting alternative reproductive tactics, at least in our study model. The low extra-pair paternity that we found is in accordance with other owl species breeding at lower densities, suggesting that density *per se* is not affecting the appearance of alternative reproductive strategies.

## Methods

### Ethics Statement

Capture, banding and blood sampling of burrowing owls were conducted under permits from the Argentinean wildlife agency (22500-4102/09), the Ethic Committee of CSIC (CEBA-EBD-11-28), and the owners of private properties.

### Study system and field procedures

The study area covers approximately 4,200 km^2^ of natural grasslands, pastures, cereal crops and urban areas near the city of Bahía Blanca (38° 43′ S 62° 16′ W; Buenos Aires, Argentina; see [21], [22]). There, we have carried out a survey program of breeding burrowing owls since 2006, accumulating 1,120 monitored nests as of 2012 (359 urban nests and 761 rural nests, most of them reoccupied between years). In our studied population, pairs are territorial and, although they can use burrows excavated by mammals for nesting, they mostly dig their own nests that are often reused from year to year. Therefore, the distribution of breeding burrowing owls is not constrained by the availability of potential nest sites, but rather by the differential susceptibility of individuals to human disturbance [21], [22]. The depth of burrow nests precluded us from gathering information on clutch size, but brood size was easily recorded since chicks often exit the nest burrow entrance during the daytime. Average brood size was 2.77 nestlings per successful breeding attempt (SD = 1.24, n = 1,253), and both parents provided parental care (authors' unpublished data). The average adult life span is less than three years [21]. The lower predation pressure faced by individuals in urban habitats together with their lower natal dispersal distances compared with that of rural ones (0.26 km vs 0.42 km on average, respectively) seem to contribute to an increase in local breeding densities [21], [22] and individual relatedness (Rodriguez-Martínez et al. in prep) in urban areas.

During the breeding seasons (late November to late February) of 2006–2012, we captured breeding individuals and chicks using bow nets and ribbon carpets placed at the entrance of active nests. Adult rural owls are more fearful of people than urban ones [21], [22], making them difficult to capture and thus reducing the number of fully-sampled rural families (see Results). All birds were marked by using a plastic ring with an individual alphanumeric code readable at a distance, and released after recording body size variables and collecting blood samples (0.2 ml). Blood samples were preserved in absolute ethanol and kept at 4°C until their processing in the laboratory. Given the diurnal behaviour of the species [21], [22], putative parents were easily identified by repeated observations (using telescopes) of the reproductive behaviour (e.g., nest attendance and defence, food provisioning) of individually marked birds across the breeding season, and were observed in their nests until the end of the reproductive period.

### Breeding densities

The diurnal activity of burrowing owls together with the flat landscape allowed us to easily locate breeding territories through the observation of pairs perched close to their burrows [21], [22]. Some pairs occupy two or more closely-spaced burrows, and thus we GPS-marked (precision ±3 m) the active nest as an estimator of the breeding territory core. We defined as urban territories those excavated by owls in private and public gardens and in spaces among houses in urbanized residential areas, but also on curbs of streets and even on large avenues in the city. Rural territories were located in the surrounding large expanses of natural grasslands and pastures devoted to wide-ranging livestock and low-intensive cereal crops, where human presence and activities are extremely low [11]. There is no clear habitat interface between urban and rural habitats, since urbanized areas are immediately surrounded by rural ones.

Because the distribution of breeding territories varied across years, estimators of conspecific densities around each active breeding territory were annually obtained using two complementary variables calculated using all breeding territories occupied each year. First, we measured the linear distance from the focal active nest to the nearest active nest (in metres). Second, we calculated an aggregation index for each active nest as its relative position within the whole distribution of the breeding population using *∑exp(-d_ij_)* (with *i≠j*), where d_ij_ was the linear distance between the active nests of breeding pairs i and j, j representing all known breeding pairs [23]. These variables were complementarily depicting the social environment of each nest at a landscape scale as well as the proximity to the closest conspecific nest.

### Genetic characterization of individuals

Genomic DNA was isolated from blood samples following a modification of the silica-based method [24]. Birds were sexed using the polymerase chain reaction (PCR) amplification of the CHD-gene [25] according to the P0/P2/P8 sexing protocol [26]. A total of 23 polymorphic microsatellites previously developed for the burrowing owl [27]–[29] were tested, individually optimized, and used to genotype all sampled individuals (Table 1). All loci were PCR amplified in two independent multiplex reactions. For each PCR sample, 6.5 µl of QIAGEN Multiplex PCR master mix, 3 µl of RNase free water (provided with the QIAGEN Multiplex PCR master mix), 1.5 µl of the primers mix (5 µl of each in a final concentration of 2 µM) and 4 µl of template DNA were used. The reaction consisted of a 5 minute denaturation step at 95°C, 32 cycles of 30 seconds at 95°C, 90 seconds at 55°C and 30 seconds at 72°C, and a final extension step of 30 minutes at 60°C. PCR products were run on 1.5% agarose gels to check for amplification and yield, and then on an ABI3100 DNA analyzer to determine DNA sizes. Genotypes were assigned, both manually and automatically, using GeneMapper 3.7 (Applied Biosystems, Foster City, CA), and all electropherograms were double-checked independently by two people.

**Table 1:** Characterization of 23 microsatellite loci used for paternity analysis in burrowing owls.

Marker	Sequence	A	N	He	Ho	PIC	HWE		
							p-value	SE	
**ATCU04 U**	AGCCATTCCCTTCAGTCTTC	3	228	0.41	0.37	0.358	0.1026	0.0051	
**ATCU04 L**	TTCATGGGTTTATGATCTGACTTC								
**ATCU06 U**	GCCATCCCTAATGCTTGTG	20	229	0.90	0.87	0.885	0.02759	0.0075	
**ATCU06 L**	GAAATGGAAGGAGGAGTGC								
**ATCU13 U**	GTTGTGAAGCGAGGGATG	7	226	0.20	0.07	0.193	<0.001	0.0000	*
**ATCU13 L**	ACCCCGAGTGCTCTAGTCAG								
**ATCU28 U**	TGGAGAGGTTTAGGGCTAGG	15	223	0.82	0.81	0.802	0.2381	0.0321	
**ATCU28 L**	CAGTGTCAGAGTCAAGACATGC								
**ATCU43 U**	GGGAGATGTTGAGGAAATCG	11	229	0.82	0.82	0.794	0.0337	0.0071	
**ATCU43 L**	GATCAGCTTGCAGCAAAGG								
**ATCU45 U**	GGGTGGACAGTTCCTCATTC	9	228	0.79	0.79	0.757	0.5714	0.0269	
**ATCU45 L**	CTACCGAGCAGTGACAGTTTG								
**BUOW04 U**	AAGACAGAGTACGGGAAG	9	229	0.78	0.83	0.748	0.9610	0.0098	
**BUOW04 L**	TCCCCTGGGAGAACTCAC								
**BUOW06 U**	GGGCTTTGGATATCAGT	4	229	0.17	0.096	0.161	0.0032	0.0010	
**BUOW06 L**	CATGAGAAAAAAAAGCAAAC								
**BUOW11U**	GGCTATAATGGGTGAGTCA	10	226	0.88	0.88	0.866	0.4653	0.015	
**BUOW11L**	GGCACTCCCTGATTGTC								
**BUOW13 U**	TCTGACCTCGCTTGCATC	3	226	0.45	0.39	0.372	<0.001	0.000	*
**BUOW13 L**	GGCCAGCTCAGTAACGTG								
**BUOW1U**	ACCACCCACAGCCACACG	6	227	0.62	0.61	0.564	0.1884	0.0135	
**BUOW1L**	AAACCCCTAACATTGTCC								
**BUOW-BM4-A01 U**	GGAAACAGCTATGACCATAGGATCTCCCAAACATTCTGGC	16	228	0.87	0.35	0.856	<0.001	0.000	*
**BUOW-BM4-A01 L**	GTTTGAATCTGGACTAGATGACCTCC								
**BUOW-BM4-A09 U**	CAGTCGGGCGTCATCAGCACTTAGGGACATGGTTTAGTGG	10	213	0.70	0.38	0.661	<0.001	0.0000	*
**BUOW-BM4-A09 L**	GTTTCCTATGAAGACCCTCAAGCCC								
**BUOW-BM4-B06 U**	GTTTCCTTATTACAAATTCACAGTG	13	228	0.77	0.81	0.7412	0.8658	0.0194	
**BUOW-BM4-B06 L**	CAGTCGGGCGTCATCAGTTCACTTTTATACATACTCCT								
**BUOW-BM4-B12 U**	GTTTCTCTTAGGTTTGGACTGGGACG	14	228	0.83	0.75	0.808	<0.001	0.0000	*
**BUOW-BM4-B12 L**	CAGTCGGGCGTCATCATGCTAGCCGTATTCCTCTACCC								
**BUOW-BM4-C12 U**	CAGTCGGGCGTCATCATCTCTCTTGCCAGGTGTTCAGG	10	227	0.80	0.77	0.772	0.0509	0.0108	
**BUOW-BM4-C12 L**	GTTTAAGCGATTTGGGAACTGGTTGG								
**BUOW-BM4-D03 U**	GTTTCAGTGAGAGTGGGTTAACAGGC	3	227	0.44	0.48	0.346	0.9289	0.0095	
**BUOW-BM4-D03 L**	CAGTCGGGCGTCATCAGGAAGATGGGTTTCAGGAACAG								
**BUOW-BM4-E11 U**	CAGTCGGGCGTCATCATCTGCTCAGTAACACAAAGCTGG	8	227	0.78	0.75	0.745	0.2889	0.0166	
**BUOW-BM4-E11 L**	GTTTATCTGGCTACAATGCTTCAGCG								
**BUOW-BM4-H06 U**	CAGTCGGGCGTCATCATTTAGGAGCAAACCAGGGAGGC	4	224	0.24	0.26	0.227	0.8984	0.0062	
**BUOW-BM4-H06 L**	GTTTGCCAGTCCAGTGAGGTGTTACG								
**BUOW-RM2-B12 U**	CAGTCGGGCGTCATCAGGCTTCCCTCTACAGCAGGTC	6	227	0.36	0.38	0.343	0.7933	0.0171	
**BUOW-RM2-B12 L**	GTTTGCTAAGCATTACCTCACATTGTTCC								
**BUOW-RM2-D04 U**	CAGTCGGGCGTCATCAGCTACCAGAATTTGGGCATGGG	2	228	0.04	0.01	0.559	<0.001	0.0000	*
**BUOW-RM2-D04 L**	GTTTACATCTGGCATTATGTTTCCCTTC								
**BUOW-RM3-1-C04 U**	GTTTGCACTGGTGCCAAACCTC	3	227	0.51	0.54	0.445	0.7859	0.0092	
**BUOW-RM3-1-C04 L**	CAGTCGGGCGTCATCACTCAGCTAATGCATCCAGTTTCC								
**BUOW-RM3-1-H08 U**	CAGTCGGGCGTCATCAGCAGAGGTTGTGCAGAGTTCAG	8	228	0.51	0.53	0.483	0.8841	0.0128	
**BUOW-RM3-1-H08 L**	GTTTATAGAGAGCGCCCAGTATGTCC								

U upper primer, L lower primer, N number of individuals successfully genotyped at each locus, A number of alleles, He expected heterozygosity, HO observed heterozygosity, PIC polymorphic information content, * HWE disequilibrium loci, after Bonferroni correction for multiple tests.

### Microsatellite variation

Deviations from Hardy-Weinberg equilibrium (HWE) and linkage disequilibrium (LD) between all pairs of loci were tested using Genepop'007 [30], applying Bonferroni's corrections for multiple tests. Probability of identity discrimination (PID) was estimated as described in Waits et al. [31] using Gimlet 1.3.3 [32]. Number of alleles, observed heterozygosity (Ho), Nei's unbiased estimates of expected heterozygosity (He), within population inbreeding coefficient (F), exclusion probability (through polymorphic information content; PIC) and frequency of null alleles were estimated at each locus as well as across loci using CERNICALIN 1.30 [33], CERVUS 3.0 [34], [35] and GENETIX 4.05.2 [36]. Standard exclusion probabilities for each locus and for the selected loci combined (Table 1) were estimated with CERVUS. Six of the 23 microsatellites explored were not at HW equilibrium (ATCU13, BUOW-BM4-A01, BUOW13, BUOW-BM4-A09, BUOW-BM4-B12, BUOW-RM2-D04), so they were excluded from parental analysis. Thus, our panel of microsatellites was reduced to 17, all of which were at linkage equilibrium (Table 1; p<0.01). PID for rural and urban birds were 1.38E^−15^ and 1.27E^−15^, respectively.

### Parentage analysis

Parentage analyses were performed in CERVUS using a maximum likelihood method. Data considered corresponded to families in which both the putative mother and the putative father were sampled, as in other situations (i.e., just the putative mother or father were sampled) we were not able to resolve parent-offspring matching with a strong level of confidence given the high levels of endogamy that we found (see below). Due to the relatively small brood size of our study population (see above) and as we were not able to detect any replacement of breeding birds within a breeding season (0 cases in 333 well-monitored nests), we included in analyses all nests with at least one offspring sampled. Nonetheless, a single chick was sampled only in 30 out of the 68 broods ultimately used for parentage analysis (see results), and in 13 of these cases brood size was 1 or 2. A nestling was considered as potentially born from extra-pair copulations or as a result of intraspecific brood parasitism when the putative father and/or mother was not among the most likely sires given by the parental pair (sexes known) analyses of CERVUS. In all of those cases, we made a posterior confirmation of the putative father and/or mother through maternity and/or paternity analyses to check for genotypic mismatches that allowed us to confidently discard paternity/maternity. Mismatch distributions between putative parents and nestlings were checked. Genotypes were simulated for 10,000 offspring, with 100% of candidate parents sampled and a total proportion of loci typed over all individuals of 0.99, assuming an inbreeding rate of 0.06% (authors' unpublished data) and a genotyping error rate estimated by CERVUS of 0.01. 8% of assignments were at the relaxed level (80%) and 92% at the stricter one (95%) [34].

### Simulations

We used Monte Carlo simulations to evaluate the probability that the spatial patterns of extra-pair paternity and intraspecific brood parasitism could have occurred by chance, only constrained by the spatial distribution of breeding sites [37], or as a consequence of increments in intraspecific densities. Thus, we generated through 1,000 randomizations the expected distributions of nearest neighbour distances and aggregation indexes by shuffling the locations of the detected cases of extra-pair paternity, intraspecific brood parasitism, and both alternative reproductive strategies among all occupied breeding territories used for parentage analysis (n = 68). 95% confidence intervals were obtained to compare them with the nearest neighbour distance and aggregation index of the territories where alternative reproductive strategies were actually observed.

## Results

**Figure 1:**
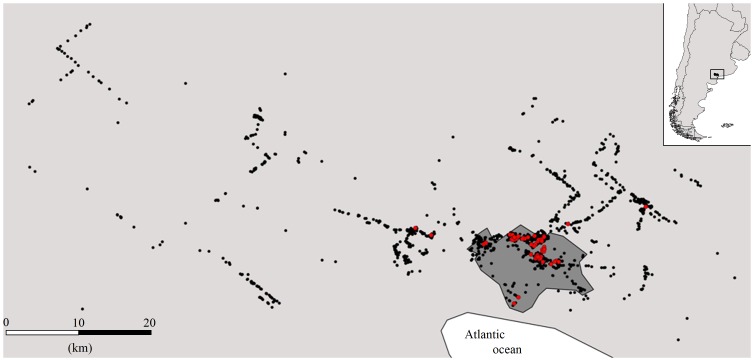
Distribution of burrowing owl nests in the study area (light grey: rural area; dark grey: urban area). Red dots show nests sampled for parentage analysis (2006: 1, 2007: 1, 2009: 15, 2010: 22, 2011: 21, 2012; 8), black dots show other active nests located during the whole study period. The aggregation of nests in the urban area is higher than observed in the figure given that many dots overlap within this area.

**Figure 2:**
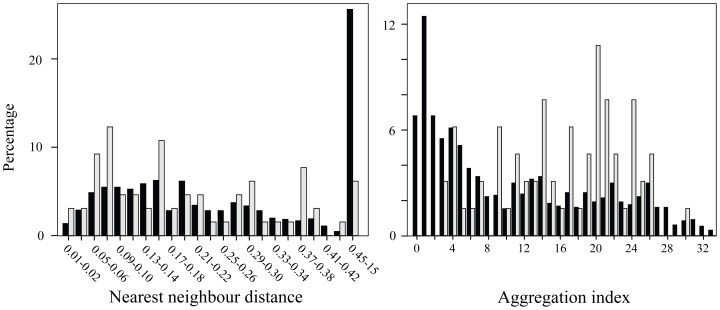
Nearest neighbour distances (in km) and aggregation indexes obtained for all occupied (black bars) and sampled (grey bars) nests of burrowing owls. Values were calculated separately for each year (see Methods).

During the breeding seasons of 2006–2012, we captured, bled and genotyped 1,107 individuals (674 chicks and 433 adults) at 565 active nests. From this total, we were able to use for analyses (see Methods) 121 chicks (plus their corresponding parents) belonging to 68 different nests (7 located in rural areas and 61 located in urban areas; Figure 1). These nests were representative of the large variability in the breeding density shown by the population, the nearest distances between active nests ranging from 0.01 to 15.07 km and the aggregation indexes ranging from 0 to 33 (Figure 2). Nests sampled for parentage analysis were slightly skewed toward high density social environments (median nearest neighbour distance, for all nests: 0.23 km, quartiles  = 0.12–0.46 km, for sampled nests: 0.16 km, quartiles  = 0.08–0.30 km; Kolmogorov-Smirnov test: Z = 1.56, p = 0.02; median aggregation index, for all nests: 7.66, quartiles = 2.32–18.34, for sampled nests: 17, quartiles = 11.01–21.29; Z = 2.93, p<0.001; Figure 2). This bias should however facilitate the detection of alternative reproductive strategies under the density hypothesis. Urban nests showed the densest breeding scenario while rural ones were located at lower densities (median nearest neighbour distances: urban nests: 0.16 km, quartiles  = 0.1–0.28 km, rural nests: 0.30 km, quartiles  = 0.16–1.49 km; Z = 5.45, p<0.001; median aggregation: urban nests: 17, quartiles  = 11.9–24.2, rural nests: 3.23, quartiles  = 1.13–14.92; Z = 12.51, p<0.001).

We detected extra-pair paternity in just one out of the 68 sampled broods (1.47%), with two chicks not genetically assigned to their putative father (mean  = 3 mismatches, SD = 1.41) in a territory located within the urban area. The resulting rate of extra-pair young was also low (1.65%, n = 121). Additionally, we found 3–7 inconsistencies in the 17 sampled loci among 7 offspring genotypes and their putative mothers (mean = 5, SD = 1.91), all of them belonging to 6 broods. Among these individuals, 5 were mismatched with their putative mothers and fathers (in 4 urban broods and 1 rural brood), while for the other 2 (in 1 urban brood) the mismatch occurred only with the putative mothers. The first cases may actually correspond to intraspecific brood parasitism, while the latter could be a consequence of quasi-parasitism (i.e., a female laying an egg in another female's nest, that egg being fertilised by the male partner at the parasitized nest). Thus, intraspecific brood parasitism could be occurring in our population in 7.35–8.82% of broods. Considering extra-pair paternity and conspecific brood parasitism together, these alternative reproductive strategies occurred at similar frequencies in rural and urban territories (14.28 and 9.83%, respectively; Yates χ^2^ = 0.14, p = 0.714).

**Figure 3:**
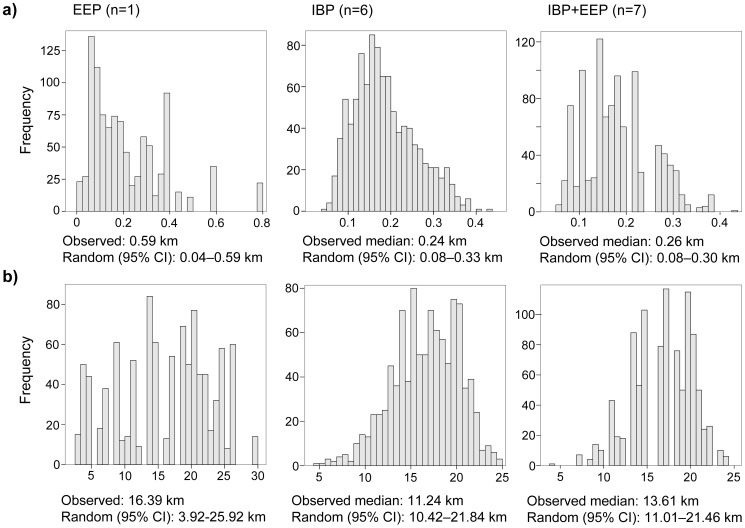
Nearest neighbour distances (a) and aggregation indexes (b) expected after randomly shuffling the number of extra-pair paternities (EEP) and intraspecific brood parasitisms (IBP) observed in the monitored population of burrowing owls. Plots are re-sampled frequency distributions. The median nearest neighbour distances and aggregation indexes of the nests where EEP and IBP were observed are provided, as well as their 95% CI expected by random chance.

For the first two years (2006–2007) we were able to sample only two complete families, so simulations to analyze the spatial distribution of alternative reproductive strategies were performed for the period 2009–2012. Simulations revealed that nests in which we observed alterations in the reproductive strategy of the species, i.e. extra-pair paternity and/or brood parasitism, were not located in more dense areas than those showing genetic monogamy (Figure 3). Indeed, these nests were at a median distance of 0.26 km to their nearest neighbours (quartiles  = 0.16–0.32 km), while their median aggregation index reached 13.61 (quartiles  = 6.54–17.97), both being within the 95% CI of the values expected by random chance (Figure 3). These results remain unchanged when considering extra-pair paternity and brood parasitism separately (Figure 3), supporting the idea that alterations in the reproductive strategy of the study species are not linked to increments in breeding densities.

## Discussion

Although most bird species were long considered monogamous [38], the widespread use of genetic markers in recent years has shown that a substantial proportion of these species are actually sexually promiscuous [39]. Indeed, alternative reproductive strategies are not rare and there is increasing evidence showing high rates of extra-pair copulations or, less commonly, intraspecific brood parasitism [40] in species considered as socially monogamous. Some authors suggest that these reproductive tactics are more frequent in particular ecological situations, such as at high breeding densities [4]. Published results are, however, conflicting, equally supporting (e.g., [41]) or refuting (e.g., [42]) the breeding density hypothesis. Here, using a large sample of broods covering a large breeding density gradient, we found that increments in density do not necessarily translate to a higher frequency in alternative breeding strategies.

The burrowing owl population studied mainly behaves as a genetically monogamous species with low extra-pair paternity rates. To our knowledge, there are no previous studies examining genetic parentage in burrowing owls. However, our results are similar to that reported for other owl species (Table 2). This low rate of extra-pair paternity among owls, and raptors in general [43]–[46], has been attributed to their low breeding densities, although other factors have also been discussed as possible causes underlying this pattern [47]. Here, we failed to find any relationship between extra-pair paternity and density. Indeed, extra-pair paternity remains as low as expected in a low density situation, suggesting that other unexplored mechanisms such as breeding synchrony [2], mate guarding [48] or male age [49] could be acting to preclude this type of reproductive strategy.

**Table 2:** Comparison of extra-pair paternity rates among owl species.

Species	Extra-pair paternity (%)	No of nestlings	No of broods	Source
Little owl *Athene noctua*	0.00	53	16	[47]
Flammulated owl *Otus flammeolus*	0.00	37	17	[68]
Tawny owl *Strix aluco*	0.70	137	37	[69]
Barn owl *Tyto alba*	0.80	211	54	[70]
Burrowing owl *Athene cunicularia*	1.47	121	68	This study
Lanyu scops owl *Otus elegans botelensis*	1.50	200	108	[71]

Intraspecific brood parasitism was reported in 234 bird species, most of them precocial, but no case was detected among owls [50]. Thus, our finding regarding intraspecific brood parasitism that may reach rates (7.35–.82%) comparable to those observed in colonial species such as European bee-eaters *Merops apiaster* (9–12%) [51], snow geese *Anser caerulescens* (5.7%) [5], common eiders *Somateria mollissima* (6%) [52], or monk parakeets *Myiopsitta monachus* (3%) [53] is intriguing. Brood parasitism is an alternative female reproductive behaviour that is poorly understood [7] and that can be evolutionarily facilitated when natal philopatry is female-biased, such that hosts and parasites are close relatives [54]. Preliminary data on natal dispersal in our study population show that females disperse at larger distances than males (female: median  = 0.46 km, quartiles  = 0.16–2.03 km, n = 54; male: median  = 0.15 km, quartiles  = 0.04–0.47 km, n = 68; Z = 1.82, p = 0.003). However, as those distances are markedly short when compared with other owl species including Tengmalm's owl *Aegolius funereus* (median = 30–56 km) [55], California spotted owl *Strix occidentalis occidentalis* (mean = 10.9 km) [56] and eastern screech owls *Otus asio* (median = 2.3 km) [57], it may be that individuals have been weakly selected to develop behaviours aimed to avoid brood parasitism [7]. The hypothesis that intraspecific brood parasitism may be favoured when individuals are close relatives [54] may be more strongly supported by the high endogamy estimated in our population (authors' unpublished data) and by observations gathered during the long-term monitoring of our individually marked population. Fledglings usually stayed with parents until a few months before breeding (when they are less than one yr old), then often mated with close relatives and bred at short distances from their natal territories. Moreover, they were never observed to engage in aggressions with neighbours (authors' unpublished data). Nonetheless, further research, including larger sample sizes and sampling neighbouring nests and mates for assessing genetic relatedness, is needed for testing this hypothesis.

The frequency at which alternative reproductive strategies occur in a population could be highly affected by breeding densities [58]. However, as our genetic results and spatial simulations suggest, changes in some of these factors alone are not enough to promote such strategies. Studies supporting the breeding density hypothesis were mostly done on songbirds (Passeriformes) [4], an order of birds in which frequencies of extra-pair paternity are relatively high [2]. However, even within this order there is no strong evidence for a general relationship between population density and extra-pair paternity across species ([59] but see [4], [60]). In this sense, our study also fails to support the density hypothesis, suggesting that the aggregation of individuals at particular sites does not necessarily promote alterations in the reproductive behaviour of individuals.

Urbanization modifies landscape structures drastically, forcing species to adapt or disappear [13]. For those species that become urban dwellers, changes in top-down or bottom-up factors that affect rates of nest predation or alter local resources [61]–[63] can prompt a variety of population level responses, including increments in densities compared with their natural counterparts [64]–[67]. Although more research is needed to properly understand the overall costs and benefits of urban invasion, our study provides strong evidence against increases in the frequency of alternative reproductive strategies despite large increases in conspecific densities in a recent urban invader [11].
